# Dysplasie fibreuse polyostotique unilatérale du membre supérieur

**DOI:** 10.11604/pamj.2014.19.81.5287

**Published:** 2014-09-25

**Authors:** Monsef Boufettal, Mohamed Azouz, Mustapha Mahfoud, Ahmed El Bardouni, Mohamed Saleh Berrada, Moradh El Yaacoubi

**Affiliations:** 1 Service de Traumatologie et Chirurgie Orthopédique, Centre Hospitalier Universitaire Avicenne, Université Mohammed V, Rabat, Maroc

**Keywords:** Dysplasie fibreuse, unilatérale, polyostotique, membre supérieur, fibrous dysplasia, unilateral, polyostotic, upper limb

## Abstract

La dysplasie fibreuse est une maladie osseuse sporadique rare d’étiologie inconnue qui représente environ 2,5% des maladies osseuses et 7% des tumeurs osseuses bénignes. Cette lésion bénigne pseudotumorale se caractérise par la présence dans l'os d'une prolifération de tissu fibreux et de tissu osseux immature dépourvu de couronne ostéoblastique. Elle peut atteindre un ou plusieurs os; l'atteinte du membre supérieur est rarement décrite, nous rapportons ici un cas rare de dysplasie fibreuse polyostotique unilatérale du membre supérieure.

## Introduction

La dysplasie fibreuse osseuse initialement décrite par Jaffe et Lichtenstein est un néoplasme bénin non transmissible du squelette osseux secondaire à une mutation activatrice du gène GNAS1 [1]. Il s'agit d'une affection tumorale bénigne, une néoformation intra-osseuse fibreuse pouvant toucher n'importe quelle segment osseux et entraîner un amincissement des corticales et donc une fragilisation osseuse [2]. Deux formes sont décrites selon le nombre de localisations: à la rare forme polyostotique s'oppose la classique forme monostotique qui représente 88% [1]. Cette affection est probablement sous-estimée, par la fréquence des formes asymptomatiques.

## Patient et observation

**Figure 1:**
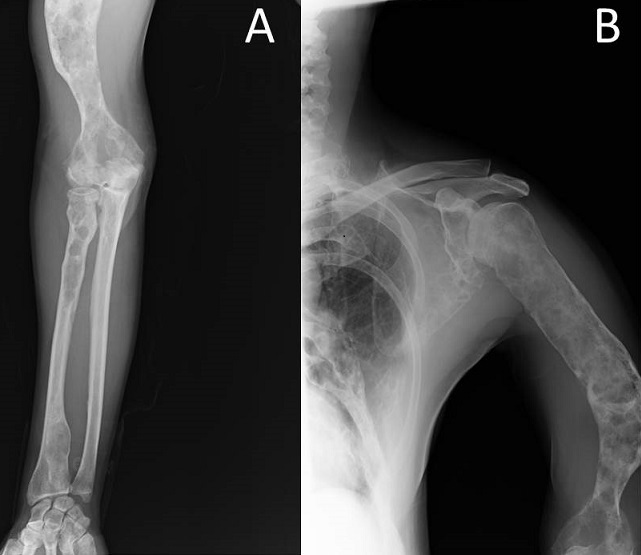
A) Radiographie de face montrant une dysplasie fibreuse du tiers proximal et distal du radius; B) Radiographie de face objectivant des lésions de dysplasie fibreuse intéressant le col de l'omoplate et la totalité de l'humérus

Il s'agit d'un patient âgé de 31 ans, admis pour des douleurs du membre supérieur gauche évoluant depuis 2 ans rebelles aux traitements antalgiques. L'examen clinique avait retrouvé une déformation du tiers distal du bras gauche avec des douleurs à la palpation du bras et à la mobilisation du coude homolatéral. Le bilan radiologique montre des images lacunaires et condensantes par endroit avec un aspect en verre dépoli. Ces lésions sont unilatérales intéressant le col de l'omoplate, la totalité de l'humérus, le tiers proximal et distal du radius alors que le cubitus était intact (Figure 1). Devant cet aspect radiologique, le diagnostic de dysplasie fibreuse dans sa forme polyostotique a été retenu. Le patient a été traité par des bisphosphonates associés à une supplémentation en vitamine D et en calcium, avec une bonne évolution. Une surveillance clinique et radiologique régulière et prolongée a été instauré devant le risque, bien qu'exceptionnel, de dégénérescence maligne de cette lésion.

## Discussion

La dysplasie fibreuse des os est une maladie rare, elle représente 7% de l'ensemble des tumeurs osseuses bénignes [1]. Elle est potentiellement sévère, à l'origine de douleurs et de déformations osseuses, de fractures, et parfois de complications neurologiques. La principale caractéristique de cette maladie est une prolifération extensive de tissu fibreux dans la moelle osseuse, due à une anomalie de différentiation des ostéoblastes [3]. L'os normal est remplacé alors par ce tissu fibreux qui comporte des petites travées osseuses réparties anormalement et qui ne sont pas bordées par des ostéoblastes. Cela entraîne l'apparition de lésions ostéolytiques, de déformations osseuses et une fragilité excessive du squelette intéressé [4]. La dysplasie fibreuse est habituellement divisée en trois formes cliniques principales: la forme monostotique, la forme polyostotique et la forme avec endocrinopathie (syndrome de Mc Cune-Albright). Les formes polyostotiques ont une symptomatologie plus marquée et se révèlent plus précocement que les formes monostotiques [2].

Le diagnostic sera fondé sur les données cliniques, radiologiques et histologiques. Les symptômes cliniques ne sont pas spécifiques, et sont à type de douleurs qui peuvent être d'origine osseuse ou articulaire, de déformations, d'inégalités de longueur des membres, de fractures et de troubles de croissance [5]. Le diagnostic sera évoqué chez les patients ayant un aspect radiologique compatible. On pensera à la dysplasie fibreuse même chez les sujets asymptomatiques. Dans les formes polyostotiques et le syndrome de Mc Cune-Albright, l'aspect radiologique est généralement suffisant pour poser le diagnostic [6]. Plusieurs aspects radiologiques peuvent être rencontrés: il peut s'agir d'une lésion lytique géodique ovalaire, de lacunes multiples, de lésion légèrement expansive, d'une opacité homogène en verre dépoli sans structures trabéculaires qui envahit la corticale, ou d'une opacité condensante [7]. Dans les formes monostotiques, l'aspect radiographique ne suffit parfois pas et le recours à la biopsie osseuse est plus fréquent, de façon à éliminer des diagnostics différentiels [6]. Certains traitements médicaux pourraient être efficaces. Au niveau des lésions osseuses, la calcitonine, un inhibiteur de l'activité des ostéoclastes, a été initialement utilisée pour réduire la résorption osseuse dans la dysplasie fibreuse polyostotique. Plus récemment, les bisphosphonates, molécules à tropisme osseux doués d'une activité antiostéoclastique, sont utilisés avec des résultats intéressants. Leur action est à la fois antalgique et recalcifiante des foyers lytiques [5, 8, 9].

Certains auteurs ont démontré l'efficacité des bisphosphonates sur la diminution du nombre de fractures et sur l'amélioration de la qualité de l'os dans les formes associées ou non à un syndrome de Mc Cune-Albright [2]. Il faut noter la place actuellement validée des bisphosphonates de nouvelle génération, tel que le pamidronate intraveineux, qui ont montré leur efficacité sur les douleurs et la reprise d'une densité osseuse satisfaisante [3]. Une supplémentation en vitamine D et calcium est également justifiée du fait de l'hyperparathyroïdie secondaire liée à la maladie elle-même, mais aussi au traitement par biphosphonates [1, 3]. L'approche chirurgicale dépendra de la localisation et de l’étendue des lésions. La majorité des fractures pathologiques survenant dans les zones de dysplasie fibreuse guérissent généralement avec des traitements conservateurs [4]. La dégénérescence sarcomateuse est une complication rare (0,4 à 1% des cas), plus fréquente dans les formes polyostotiques. La dégénérescence se fait en ostéosarcome, en fibrosarcome, plus rarement en chondrosarcome ou en histiocytofibrome malin [1, 5, 6]. Le pronostic dépend de l'importance de l'atteinte du squelette et des manifestations extrasquelettiques. En règle générale, les formes limitées ont un bon pronostic indépendamment de l’âge de découverte. À l'opposé, les formes très étendues, survenant tôt dans la vie, entraînent des fractures et des déformations se poursuivant à l’âge adulte, avec un mauvais pronostic fonctionnel [5, 10].

## Conclusion

La dysplasie fibreuse est une pathologie rare, pouvant se manifester sous différentes formes, se caractérisant par une variabilité des signes cliniques et radiologiques. Son diagnostic précoce même au stade asymptomatique permet une meilleure prise en charge thérapeutique et peut prévenir la dégénérescence sarcomateuse.
